# Supplementary figures and images for: Post-operative outcomes in Indigenous patients in North America and Oceania: A systematic review and meta-analysis
Source: PLOS Glob Public Health. 2023 Aug 16;3(8):e0001805. doi: 10.1371/journal.pgph.0001805 (PMC10431673; doi:10.1371/journal.pgph.0001805)

**S1 Appendix: PRISMA-SCr and MOOSE Checklists**


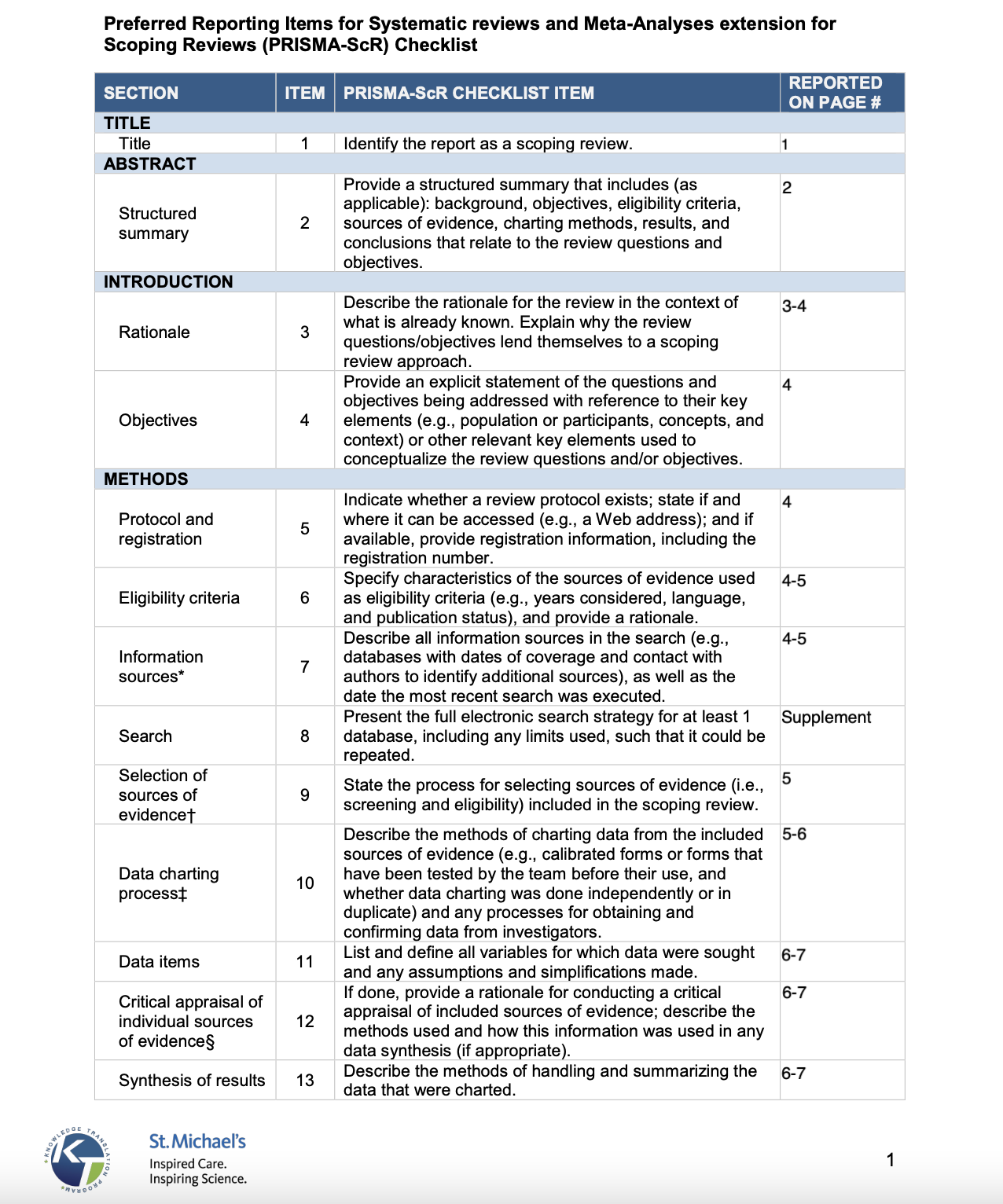


**
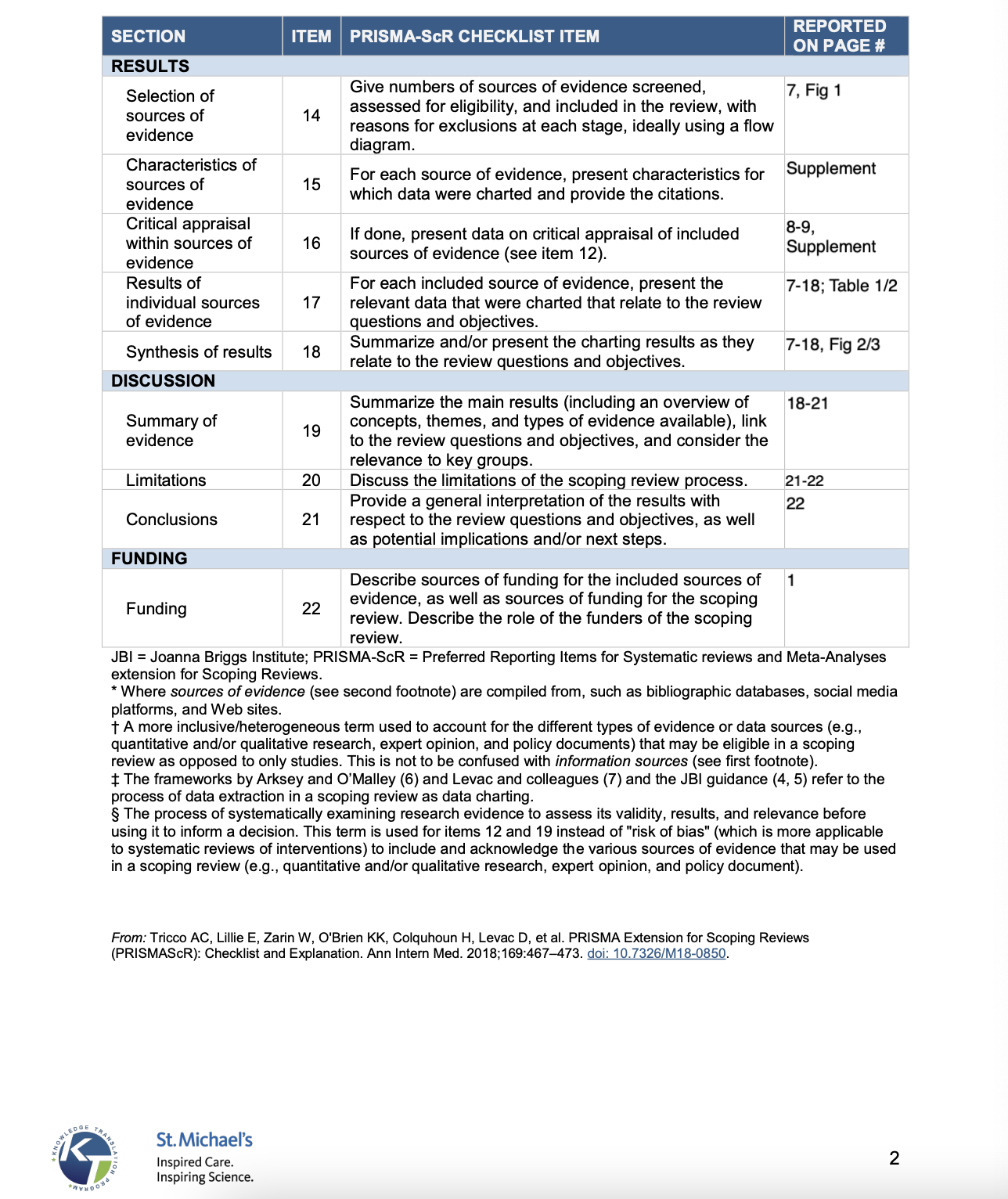
**

**
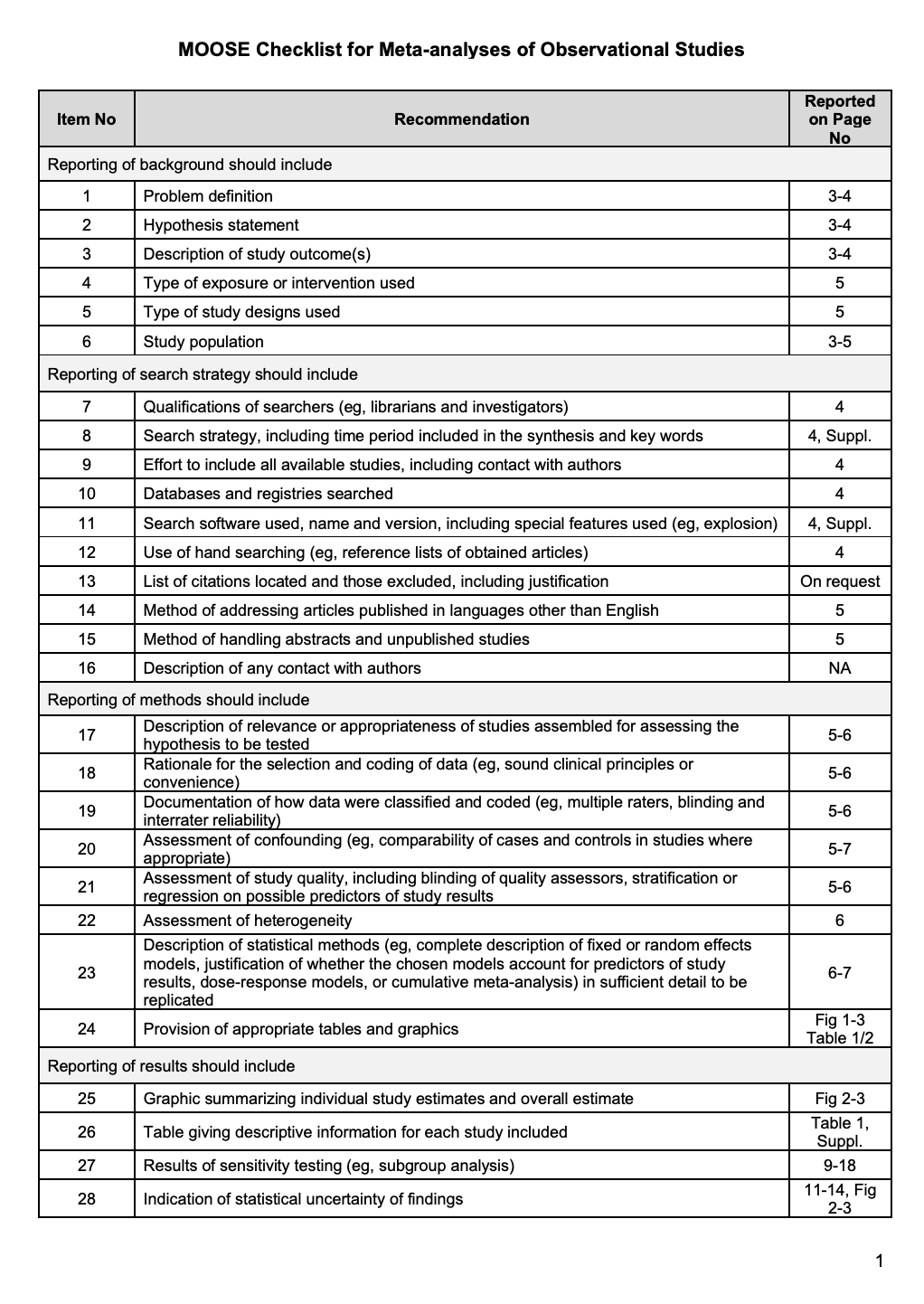
**

**
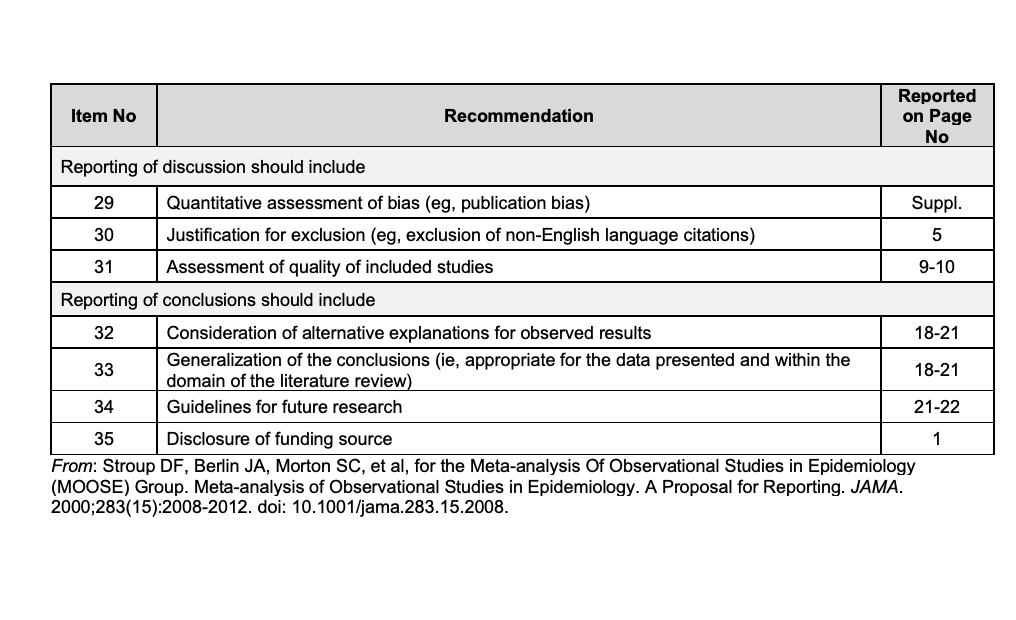
**

Supplement: S1 Fig — (DOCX) [file pgph.0001805.s001.docx]

**S2 Fig: Quality and risk of bias assessment of included studies**


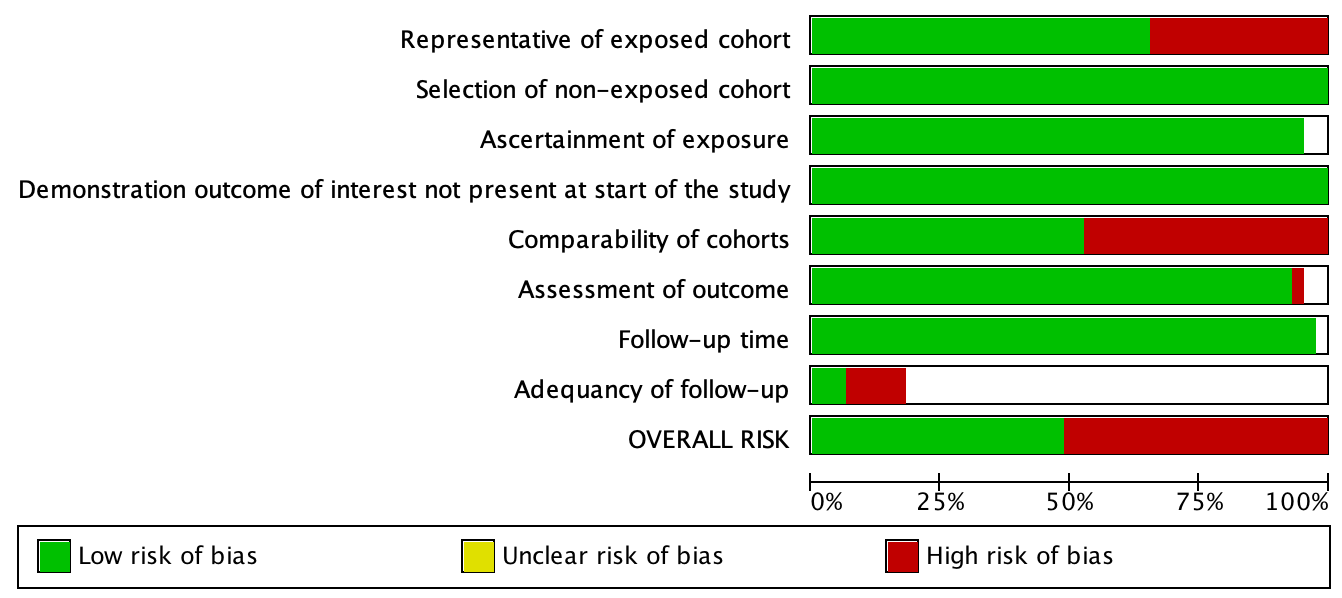

Supplement: S2 Fig — (DOCX) [file pgph.0001805.s002.docx]
