# Supplementary material for: Post-operative outcomes in Indigenous patients in North America and Oceania: A systematic review and meta-analysis
Source: PLOS Glob Public Health. 2023 Aug 16;3(8):e0001805. doi: 10.1371/journal.pgph.0001805 (PMC10431673; doi:10.1371/journal.pgph.0001805)
Supplement: S3 Fig — (DOCX) [file pgph.0001805.s003.docx]

**S3 Fig: Funnel plots for publication bias assessment**

| **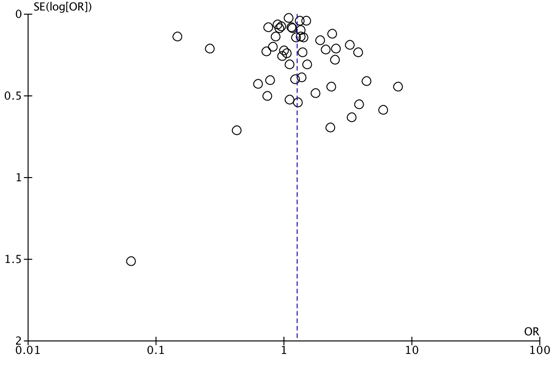** | **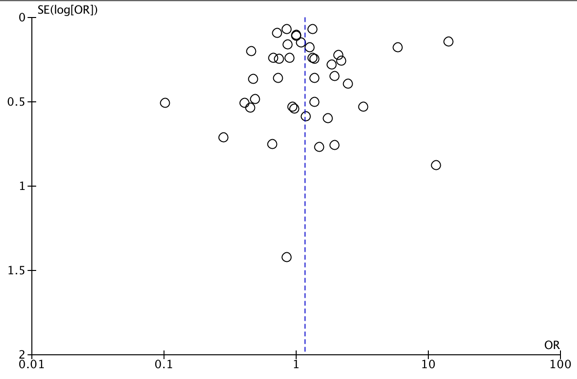** |
| --- | --- |
| Overall Morbidity | Overall Mortality |
| **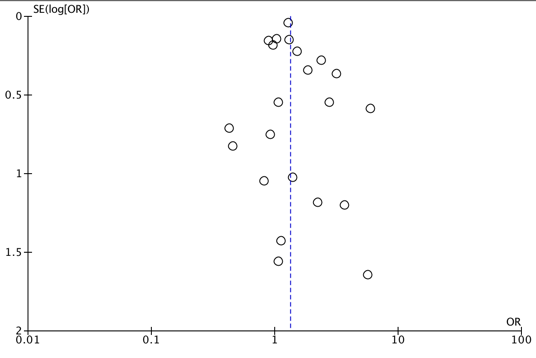** | 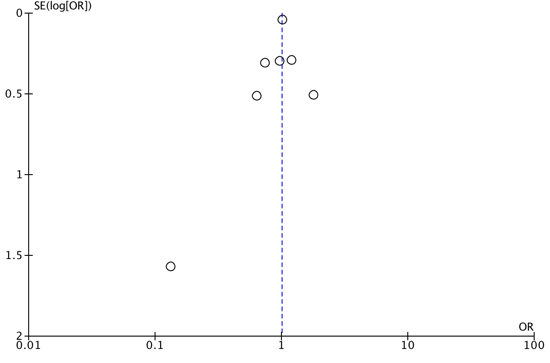 |
| Surgical Infections | Systemic Infections |
| 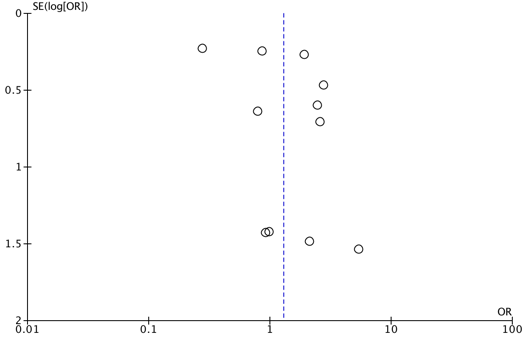 | 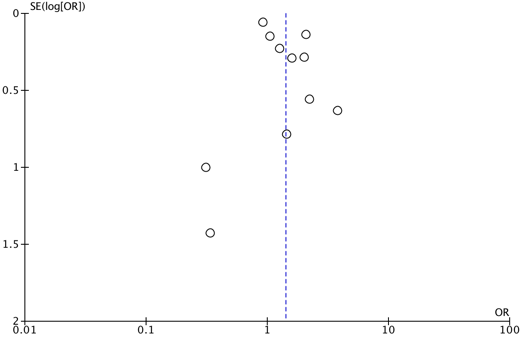 |
| Cardiovascular Complications | Pulmonary Complications |
| 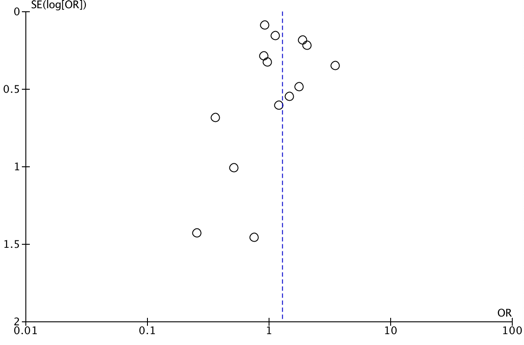 | **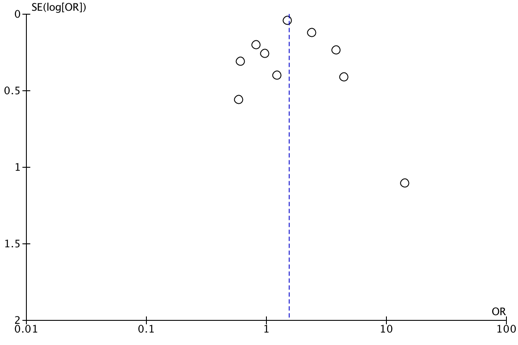** |
| Hematologic/Thromboembolic Complications | Immunologic Complications |
| **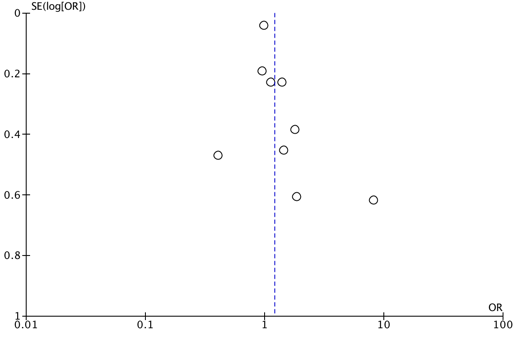** | 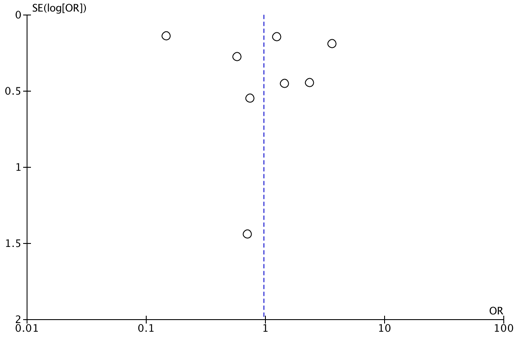 |
| Genitourinary Complications | Procedural Complications |
| **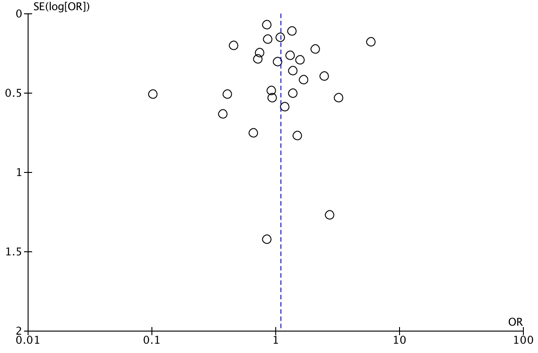** | **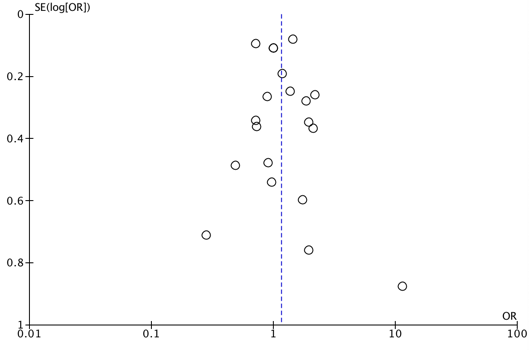** |
| <30 Day Mortality | >30 Day Mortality |
| 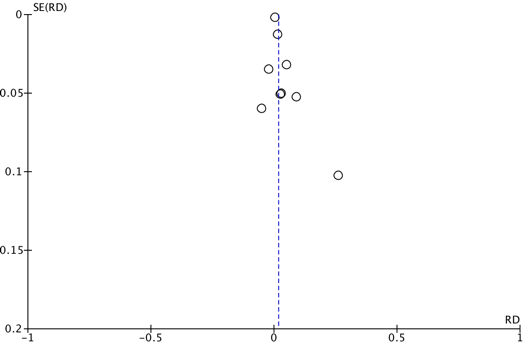 | *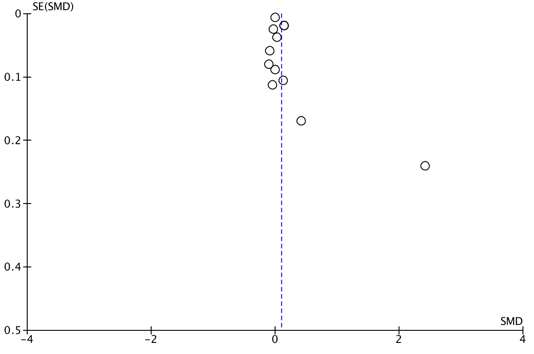* |
| Reoperation | Readmission |
| **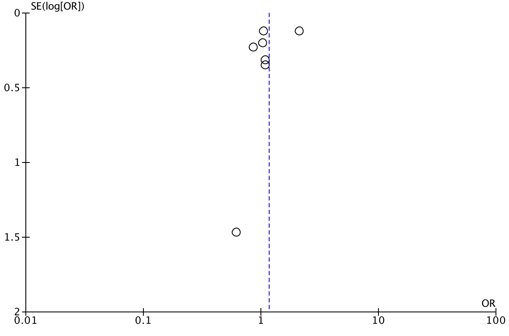** |  |
| Length of Hospital Stay |  |
